# Prospective cohort study of induction of labor: Indications, outcome and postpartum hemorrhage

**DOI:** 10.18332/ejm/142782

**Published:** 2021-11-11

**Authors:** Bid Kumar, Sujatha Kumari, Stephen Hughes, Stuart Savill

**Affiliations:** 1 Department of Obstetrics and Gynaecology, Wrexham Maelor, Hospital, Wrexham, United Kingdom; 2 Department of Obstetrics and Gynaecology, Ysbyty Gwynedd Hospital, Bangor, United Kingdom; 3 North Wales Clinical Research Centre, Wrexham Technology Park, Wrexham, United Kingdom

**Keywords:** Bishop’s score, caesarean section, postpartum hemorrhage, gravimetric method, induction of labor, induction-delivery interval, instrumental vaginal birth

## Abstract

**INTRODUCTION:**

This study was undertaken because of the increasing rate of induction of labor (IOL) in our hospital and its associated higher caesarean section (CS) rates. The objective was to ascertain the incidence, indications, methods, outcome, and complications of IOL, in particular postpartum hemorrhage.

**METHODS:**

This was a prospective observational cohort study of women who underwent IOL in a medium-sized district general hospital. Blood loss was measured by the gravimetric method and correlated to postpartum hemoglobin level within 48 hours of birth.

**RESULTS:**

A total of 445 women needed IOL (incidence 33%). Common indications were: small for gestational age (SGA) or fetal growth restriction (FGR) (18%), spontaneous rupture of membrane (17%), reduced fetal movement (16%), prolonged pregnancy (15%), and diabetes (13%). In all, 67% women achieved spontaneous vaginal delivery and 18% underwent caesarean section. With regard to blood loss, 62 women (14%) had postpartum hemorrhage (PPH) of >1000 mL and 22 women (4.9%) had a blood loss >1500 mL. The caesarean section rate was higher than the overall emergency caesarean section rate in that year. Incidence of PPH in this cohort was higher than the normal incidence.

**CONCLUSIONS:**

Increasing trend of induction of labor (IOL) is due to the changing clinical policy on management of small for gestational age babies, spontaneous rupture of membrane, reduced fetal movement and other complications of pregnancy. There is conflicting evidence on the effect of IOL on caesarean section rates. IOL is a risk factor for PPH. Accurate measurement of blood loss is essential in detecting a fall in hemoglobin which in turn helps in the appropriate management of PPH.

## INTRODUCTION

The rate of induction of labor (IOL) has steadily increased since 1980 in the United Kingdom and around the world, influenced by pre-existing medical conditions, advanced age and changing fetal indications for IOL.

The National Institute for Health and Care Excellence (NICE) recommends that at the 38th week antenatal visit, all women should be offered information about the risks associated with pregnancy that lasts longer than 42 weeks and offer IOL between 41–42 weeks^1^.

The Royal College of Obstetrics and Gynaecology (RCOG) and NICE guidelines recommend earlier induction of labor for various maternal and fetal indications. In our hospital after the launch of the Growth Assessment Protocol (GAP) with customized growth chart, we have noted an increased detection of pregnancies with small for gestational age fetus^2^. According to our policy, such women are offered IOL at or after 37 weeks of gestation depending on clinical circumstances.

Despite evidence to the contrary, there are many who feel that increasing incidence of IOL leads to increasing incidence of CS. IOL can place more strain on maternity services due to prolonged length of stay in hospital and heightened fetal monitoring^3^.

As per the National Maternity and Perinatal Audit Report (NMPA) 2019, the rate of IOL in Britain in women with term singleton pregnancy is 29.6%, whereas in Wales, it is 30%. The corresponding figures for overall rate of caesarean birth were 25.8% and 24%^4^.

The objective of this study was to ascertain the incidence, indications, methods and mode of birth, duration of labor, and complications including PPH, in women undergoing IOL.

Our interest in PPH stemmed from local concerns about a markedly higher rate of PPH in women undergoing IOL. To rule out the possibility of overestimation, we tested whether our method of measuring blood loss was accurate enough. We also looked at the correlation between the measured blood loss (MBL) and reduction in hemoglobin level.

## METHODS

This study was carried out over six months from 1 May 2018 to 31 October 2018 and was registered (Reg. No. 18/036) with the Department of Clinical Effectiveness and Audit in Wrexham Maelor Hospital in Betsi Cadwaladr University Health Board (BCUHB), North Wales, United Kingdom. During this period, 514 women had an appointment for IOL. Spontaneous labor ensued in 61 women before their scheduled appointment for IOL. Clinical records of 8 women could not be accessed and it was uncertain whether they underwent IOL or augmentation of labor. Hence, they were excluded. Therefore, 445 women were included in this study. We collected data prospectively on a weekly basis on a pre-designed proforma.

Primary PPH was defined according to tradition as blood loss of ≥500 mL within 24 hours of birth. Blood loss was calculated by the gravimetric method and was statistically correlated to the postpartum hemoglobin concentration obtained within 24–48 hours after birth.

For vaginal birth, a dedicated birthing drape sheet incorporating a graduated plastic pouch was used. All linen and swabs soaked with blood were weighed. Spillage of blood on to the bed or floor was mopped up with swabs and weighed. The dry weight, of linen, swabs and gauze pieces, was then deducted from the wet weight. Midwives and healthcare assistants are trained to carry out this gravimetric method. The volume of amniotic fluid in vaginal birth is usually not significant as most often most of the amniotic fluid will have been already drained.

For CS we use dedicated sterile surgical drapes incorporated with graduated plastic pouches on all sides around the incision window where all the fluid effluent is collected for measurement of volume. In a similar process like vaginal birth, all linen, swabs and gauze pieces are weighed. For our study, 1g weight of blood was equated with 1 mL blood volume^5,6^.

Statistical analysis for this clinical study was carried out using SPSS for Windows, version 26. Initial testing for normal distribution was carried out on all sample populations, with results being measured against the Kolmogorov–Smirnov test, with data being classified as normally distributed if p≥0.05.

Data were analyzed employing the Spearman’s rank sum and Friedman statistical tests, adopting a 5% level of significance. All data are presented as medians with interquartile range (IQR) or means with standard deviation.

## RESULTS

### Incidence, body mass index (BMI) and parity

Total number of births in our hospital during the six months study period was 1338 out of which 445 were induced; 35% of these women were obese (BMI >30 kg/m^2^); 218 women were nulliparous and 227 were parous. The incidence of IOL during the period of this cohort study was 33%. Incidence was 37% in nulliparous and 30% in multiparous women.

### Indications for IOL

The most common indication was fetal growth concerns like SGA, FGR on its own or in combination with other secondary indications. Eighty-two (18%) women were induced for a primary indication of SGA or FGR. Seventy-eight (17%) women underwent IOL for prolonged spontaneous rupture of membranes (SROM). Seventy-two (16%) for reduced fetal movements as primary indication or in combination with other factors. Sixty-seven (15%) women were induced due to prolonged pregnancy (40+12 weeks). Fifty-seven (13%) underwent IOL for maternal diabetes and 28 (6%) for large for gestational age (LGA) fetus.

### Gestational age at IOL

In all, 432 women (97%) had IOL after 37 completed weeks of gestation of which 253 women were induced between 37+0 weeks and 39+6 weeks, 87 were induced between 40 to 40+6 weeks, 90 were induced between 41 to 41+6 weeks and 2 at 42 weeks of gestation.

Thirteen (3%) women had IOL before 37 weeks, out of whom 5 were between 35 and 35+6 weeks and 8 were between 36+0 and 36+6 weeks gestation. Eight of these preterm IOL were undertaken due to preterm pre-labor rupture of membrane, 3 were undertaken due to fetal growth restriction and 2 were multiple gestation. Two out of 13 women had caesarean section.

### Methods used for IOL

In our unit we use Propess^®^ (10 mg dinoprostone or prostaglandin E2) 24 hours controlled release pessary as initial method of induction if the Bishop score (BS) is unfavorable or the cervix is not suitable for artificial rupture of membranes (ARM). After one dose of Propess^®^, if ARM is not feasible, we consider the use of Prostin^®^ (3 mg dinoprostone or prostaglandin E2) vaginal pessary, used 6 hourly. In women in whom the cervix becomes favorable after use of prostaglandin E2, we perform ARM and then, depending on circumstances, oxytocin infusion is started. Women with pre-labor rupture of membranes are either induced with one dose of Prostin^®^ followed by oxytocin or directly with oxytocin depending on BS.

Of 445 women, 335 had 10 mg dinoprostone pessary as the initial method of IOL (2 women were given 2 doses each, one followed by another in 24 hours). Of these 335 women 211 were induced with 10 mg dinoprostone and did not need any further prostaglandin so were transferred to the labor ward. There were 124 women (37%) who needed further prostaglandin in the form of 3 mg dinoprostone vaginal tablet, thus 46 women needed 1 dose, 46 women needed 2 doses, 31 women needed 3 doses and 1 woman needed 5 additional doses.

In all, 53 women had 3 mg dinoprostone as initial method of IOL, 34 had artificial rupture of membranes (ARM) and 23 had oxytocin as initial method of IOL.

Overall, 236 (53%) women needed oxytocin for IOL, including 23 who had it as primary method of IOL after ruptured membranes. In our department, oxytocin infusion for IOL is started at 1 mU/min, increased every 30 mins until 4 to 5 uterine contractions occur every 10 mins. If necessary, the rate is increased to a maximum dose of 32 mU/min, but in multiparous women and women with previous CS we tend to keep the maximum infusion rate to a much lower dose. The oxytocin infusion is prepared by mixing 1 mL (10 units) of oxytocin in 49 mL of normal saline and given through a syringe pump.

### Induction to delivery interval

On average, it took nulliparous women 39 hours and 32 minutes to deliver after starting IOL, whereas in multiparous women interval between IOL to delivery was 29 hours and 48 minutes. This was influenced by BS at initiation, as shown in Table 1.

**Table 1:** Implication of Bishops score (BS) at start of induction of labor (IOL) on duration of labor and caesarean section (CS) rate

	*BS at start*				
	*0*	*1*	*2*	*3*	*≥4*
Total number of women	30	58	89	85	124
Nulliparous	24	38	36	41	56
Multiparous	6	20	53	44	68
Time between IOL and delivery	62 h 44 min	50 h 41 min	35 h 6 min	41 h	27 h 17 min
CS, n (%)	8 (26.7)	15 (25.8)	20 (22.4)	13 (15.2)	21 (16.9)

Note: 57 women had either ARM or oxytocin as the method for IOL. Practically none of these women needed assessment of Bishop score. There were a further 2 women for whom a Bishop score was not documented, so the total number of women with Bishop score was 386.

### Delay in starting elective induction of labor

Of the 445 IOL women, 35 were deferred from the scheduled appointment time. Of these, 17 had IOL on the same day but later than planned, 14 women had a delay of 1 day and 4 women had a delay of 2 days. On an average the delay was about 5 hours during the study period (range: 0–48 h)

### Mode of delivery

Of the 445 women who underwent IOL, 296 had spontaneous vaginal birth (67%), 67 had assisted vaginal birth (15%) and 82 had caesarean section (18%). There were 227 nulliparous and 218 multiparous women. In the nulliparous group, 116 (51%) had spontaneous vaginal birth, 59 (26%) had assisted vaginal birth and 52 had caesarean section (23%). The corresponding figures amongst multiparous women were 180 (82%), 8 (4%) and 30 (14%), respectively. Mode of delivery was influenced by the BS at initiation of IOL, as shown in Table 1.

Understandably, the rate of CS varied with the indication for IOL, as shown in Table 2.

**Table 2:** Caesarean section rate amongst nulliparous and multiparous women based on indication for induction of labor

*Indication for IOL*	*Nulliparous CS/total IOL n/N (%)*	*Multiparous CS/total IOL n/N (%)*
Study overall (n=445)	52/227 (23)	30/218 (14)
SGA/FGR (n=82)	6/26 (23)	10/56 (18)
Prolonged rupture of membrane (n=78)	10/54 (17)	3/24 (12)
Decreased fetal movements (n=62)	10/38 (26)	2/34 (3)
Prolonged pregnancy (n=67)	8/42 (19)	5/25 (20)
Maternal diabetes (n=57)	13/29 (45)	6/28 (14)
Large for gestational age (n=28)	5/15 (33)	4/13 (23)

CS: caesarean section. IOL: induction of labor. SGA: small for gestational age. FGR: fetal growth restriction.

### Indications for caesarean section

Amongst the 82 CS women in this cohort, 32 were carried out for fetal distress, 9 for failed IOL, 26 for failure to progress, 5 each for failed instrumental delivery and maternal request, one for scar tenderness, and 4 for unstable lie or malpresentation.

### Postpartum hemorrhage

A total of 183 women (41%) from this cohort had PPH with a measured blood loss (MBL) of ≥500 mL. Blood loss of 500–1000 mL was defined as minor PPH, and blood loss of >1000 mL was defined as major. Major PPH was further classified into moderate (1001–2000 mL) and severe (>2000 mL). Major PPH was seen in 63 women (14%) in this study. We distributed blood loss into five categories according to mode of delivery as shown in Table 3.

**Table 3:** Mode of delivery related to the category of postpartum hemorrhage volume (mL)

*Mode of delivery*	*500–1000 mL n/N (%)*	*1001–1499 mL n/N (%)*	*1500–2000 mL n/N (%)*	*2001–2499 mL n/N (%)*	*≥2500 mL n/N (%)*
Caesarean section (N=82)	35/82 (43)	15/82 (18)	5/82 (6)	3/82 (4)	2/82 (2)
Assisted vaginal delivery (N=67)	24/67 (36)	12/67 (18)	4/67 (6)	1/67 (1.4)	1/67 (1.4)
Spontaneous vaginal delivery (N=296)	61/296 (21)	13/296 (4)	5/296 (1.6)	1/296 (0.3)	1/296 (0.3)
Total	120	40	14	5	4

Percentages in rows do not add up to 100% because women who lost <500 mL of blood have not been included.

Statistical calculations were undertaken to find out: a) if there was significant correlation (Spearman’s rank sum test) between the measured blood loss (MBL) and fall in hemoglobin concentration in any or all of the three categories shown in Table 4, and b) whether the differences (Friedman test) between the fall in hemoglobin concentration in those three categories were significant. Tests of normality demonstrated that MBL was not distributed normally. Hence data are presented as median values with their interquartile range in Table 4.

**Table 4:** Relation of category of measured postpartum hemorrhage volume (mL) with mean hemoglobin concentration and mean difference in fall of hemoglobin concentration

	*500–1000 mL Category 1*	*1001–1499 mL Category 2*	*≥1500 mL Category 3*
Number of women	120	40	23
Median blood loss (mL) (IQR)	700 (257)	1175 (251)	1900 (263)
Predelivery minus postdelivery hemoglobin level (g/L), mean±SD	16.11±11.71	23.49±13.58	32.52±14.18
Postpartum hemoglobin level (g/L), mean±SD	102.3±12.506	95.4±12.42	88.04±12.70
Number of women transfused blood	0	2	5

Hemoglobin concentration and the difference or fall in hemoglobin concentration in each category were distributed normally and hence a mean and standard deviation for these factors are shown in Table 4.

Spearman’s rank sum test (non-parametric test) produced a significant correlation value of -0.455 (for 2-tailed test correlation is significant at 0.01 level) for relation between MBL and fall in hemoglobin concentration. Applying the Friedman test, the difference between the drop in hemoglobin levels in the three categories of blood loss was also found to be significant with p=0.001. Upon post hoc analysis significant changes were found between: Category 1 vs Category 2 (p=0.002), Category 2 vs Category 3 (p=0.023), and Category 1 vs Category 3 (p=0.001).

Obstetric anal sphincter injury (OASI) occurred in 2.9% of women following IOL in this cohort. This rate is comparable to the rate stated in RCOG’s Green top guideline and slightly lower than the overall rate reported in the UK national audit report^4,7^. However, the rate in this cohort was higher than the overall rate in mothers giving birth (1.7%) during this study period in our hospital. Third degree perineal tear occurred in 11 women, 7 with normal delivery and 4 associated with instrumental delivery. Fourth degree occurred in 2 women, both of whom had forceps delivery. Table 5 shows a comparison of the outcome measures with the overall number of women giving birth in our hospital during the six months when this cohort study was undertaken.

**Table 5:** Comparison of outcome measures in the study cohort with the overall number of women giving birth during the same six-month period

	*Total (N=1338) n (%)*	*In this study cohort (N=445) n (%)*
Spontaneous vaginal birth	832 (62)	296 (66)
Nulliparous	586 (44)	218 (49)
Multiparous	752 (56)	227 (51)
Instrumental	157 (12)	67 (15)
Emergency caesarean section	205 (15)	82 (18)
OASI	24 (1.7)	13 (2.9)
Shoulder dystocia	15 (1.1)	5 (1.1)
PPH ≥1500 mL	57 (4.3)	23 (5.1)
Epidural	232 (17)	116 (26)

OASI: obstetric anal sphincter injury. PPH: postpartum hemorrhage.

## DISCUSSION

Rate of IOL in this cohort was slightly higher than the overall UK induction rate of 29.6% and Welsh incidence of 30.1% during 2016–2017^4^.

This cohort had a BMI of ≥30 (kg/m^2^), which is significantly higher than the obesity rate of 22 % in the UK national audit^4,8^. Medical disorders, like gestational hypertension, diabetes in pregnancy, fetal growth concerns like SGA or LGA, and PPH, may have an association with this high incidence of obesity in our population.

For SROM before 34 weeks of gestation, we offered IOL after 34–35 weeks if labor did not start spontaneously. For women with SROM after 34 completed weeks, we would normally offer IOL after 24 hours if spontaneous labor did not start. However, since this cohort study, the RCOG^9^ has published revised guidelines which recommend IOL after 37 weeks for women with preterm SROM. Had we had the advantage of this latest guidance, we could have delayed IOL in some women and in that case quite likely many women would have labored spontaneously. We note that our second commonest indication for IOL was SROM.

The vast majority of women in this cohort were induced after 37completed weeks of gestation facilitated by the effective service we provide in our Obstetric Day Assessment unit.

Additional prostaglandin, besides the primary dose of 10 mg dinoprostone was needed in just over a third of the women. This shows that contrary to common belief, one dose of sustained release 10 mg dinoprostone is not always enough to induce labor and further doses of prostaglandin have to be considered judiciously, particularly in women with previous CS. We did not encounter any complications as a result of such additional doses.

Due to the busy wards, elective IOL was sometimes deferred, usually due to staffing issues, excessive workload, and other emergencies. On occasions, women with favorable cervix suitable for ARM had to wait before being transferred to the labor ward for ARM.

The proportion of assisted vaginal delivery in this cohort was slightly higher than the overall rate of 12% assisted vaginal births in our hospital during the study period and the national rate of 12.5–13% in the UK^4,10^.

In this cohort, the CS rate was higher than the overall emergency CS rate in our hospital during this study period of six months. In our hospital in year 2018, the overall CS rate was 25.23%, of which emergency CS was 14.18%. The national perinatal audit of 2016–2017 reported an emergency CS rate of 14.5%^4^. The effect of IOL on the rate of CS seems to be controversial, uncertain and undecided subject, as evident from contradictory views in numerous publications on the subject^11-20^.

Of the 82 women who had CS, 9 were due to failed induction, which translates to a failed induction rate of 2% of all inductions in this cohort.

The spontaneous vaginal delivery (SVD) rate was slightly higher than the overall rate of SVD in our hospital during the study period and the UK national rate for SVD, both of which were 62% (NMPA, 2019). Not surprisingly, the initial BS was directly related to the length of labor and CS rate. CS rate was much higher in women with an initial BS of zero when compared with women whose initial BS ≥4. The same phenomenon was also noted in another study^21^.

Eighty-two women underwent IOL for SGA fetus and/or FGR, of whom 26 were nulliparous and 56 were multiparous. In this sub-group of women with SGA/FGR babies, 6 nulliparous women (23% of nulliparous) and 10 multiparous women (18% of multiparous) delivered by CS. In this cohort, 19% women with SGA/FGR were delivered by CS, which was similar to the rate of CS in this cohort. A previous study showed that term patients undergoing IOL with SGA fetus are as likely to achieve a vaginal delivery as patients with non-SGA fetus^22^.

We noted that the CS rate was significantly higher among nulliparous women who had IOL for diabetes. A recent study concluded that IOL for diabetes is not associated with increased risk of CS and should not be avoided in an attempt to minimize risk of CS^23^.

CS rate was higher in both nulliparous and multiparous women following IOL for LGA babies. Higher rate of CS in women with LGA babies has been reported in another study^24^.

In this cohort, 41% had PPH (blood loss ≥500 mL), of which 27% had minor PPH and 14% had major PPH; 5.1% women had a PPH >1500 mL, which is significantly higher than the overall UK national rate of 2.9%^4^. In comparison, overall, 4.3% of women in our hospital had PPH ≥1500 mL during this study period.

Seven women received blood transfusion following PPH. Hemoglobin concentration in these women 24–48 hours after birth were between 66 and 76 g/L in six women, and 87 g/L in the rest.

A previous study has shown that IOL, regardless of the method used, is associated with a higher risk of PPH than spontaneous labor even in low-risk women^25^. Another study concluded that use of oxytocin during labor appears to be an independent risk factor for severe PPH^26^.

It is widely known that visual estimate of blood loss is inaccurate^27,28^. Like other studies, our study confirmed the efficacy of the gravimetric method for measuring blood loss^29-33^. MBL significantly correlated with fall in hemoglobin concentration in all the three categories of PPH. As suggested in other studies, 6 of the 7 women who needed blood transfusion had a postpartum hemoglobin concentration of <80 g/L^34^.

## CONCLUSIONS

The increasing rate of IOL is, to a large extent, related to the latest guidance on management of SGA/FGR babies as well as to modern management of pregnancy complications. There is conflicting evidence on whether IOL leads to increased rates of CS. In the present cohort, the CS rate was higher in women who underwent IOL. Multiple doses of prostaglandin for IOL, as used in this study, when used judiciously, did not cause any harm. Additional doses of uterotonics must be used judiciously, particularly in the case of women with previous CS. As expected, the initial BS directly related to length of labor and mode of birth. CS rate was significantly higher in nulliparous women who had IOL for maternal diabetes and women who had IOL for LGA babies.

IOL seems to be a risk factor for minor and major PPH. The gravimetric method of measuring blood loss is a satisfactory technique and correlates significantly with decrease in hemoglobin concentration. Our study also showed that instrumental vaginal birth had a significant association with 3rd or 4th degree perineal tears^35^. Nearly half of all such tears occurred following instrumental vaginal birth. There was an increased use of epidural for pain relief in labor by women undergoing IOL.

## Data Availability

The data supporting this research are available from the authors on reasonable request.
